# Reaction of selected carbohydrate aldehydes with benzylmagnesium halides: benzyl versus *o*-tolyl rearrangement

**DOI:** 10.3762/bjoc.10.202

**Published:** 2014-08-20

**Authors:** Maroš Bella, Bohumil Steiner, Vratislav Langer, Miroslav Koóš

**Affiliations:** 1 Institute of Chemistry, Center for Glycomics, Slovak Academy of Sciences, Dúbravská cesta 9, 845 38 Bratislava, Slovakia; 2 Environmental Inorganic Chemistry, Department of Chemical and Biological Engineering, Chalmers University of Technology, 412 96 Göteborg, Sweden

**Keywords:** aldehyde, benzyl versus *o*-tolyl, carbohydrate, Grignard reaction, rearrangement, X-ray crystallography

## Abstract

The Grignard reaction of 2,3-*O*-isopropylidene-α-D-*lyxo*-pentodialdo-1,4-furanoside and benzylmagnesium chloride (or bromide) afforded a non-separable mixture of diastereomeric benzyl carbinols and diastereomeric *o*-tolyl carbinols. The latter resulted from an unexpected benzyl to *o*-tolyl rearrangement. The proportion of benzyl versus *o*-tolyl derivatives depended on the reaction conditions. Benzylmagnesium chloride afforded predominantly *o*-tolyl carbinols while the application of benzylmagnesium bromide led preferably to the *o*-tolyl carbinols only when used in excess or at higher temperatures. The structures of the benzyl and *o*-tolyl derivatives were confirmed unambiguously by NMR spectral data and X-ray crystallographic analysis of their 5-ketone analogues obtained by oxidation of the corresponding mixture of diastereomeric carbinols. A possible mechanism for the Grignard reaction leading to the benzyl→*o*-tolyl rearrangement is also proposed.

## Introduction

One of the most popular synthetic routes leading to the formation of simple alkyl or aryl branched-chain sugars involves the addition of Grignard reagents. In this regard, a wide variety of Grignard reagents have been added to the free or masked (as hemiacetal) carbonyl functionalities present in the molecule of a suitable fully *O*-protected saccharide, thereby making possible the preparation of a series of useful carbohydrate derivatives [1–8]. Despite the demonstrable advantages of the Grignard reaction, there remain, in addition to the recognised drawbacks, some new unexpected impediments limiting its application in the synthesis of branched carbohydrates.

**Scheme 1:**
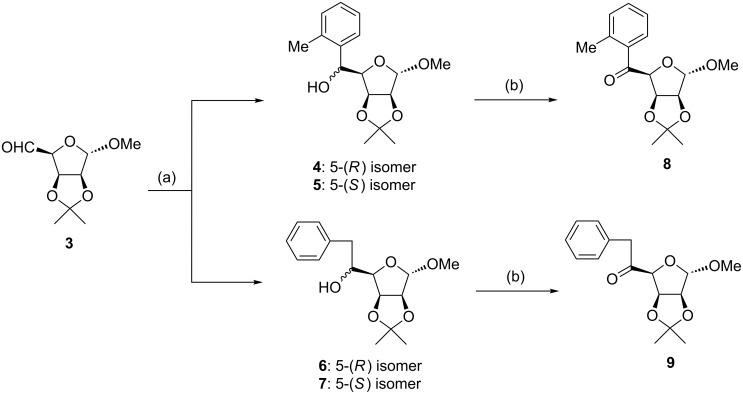
Grignard reaction of aldehyde **3** and oxidation of the resulting mixture of alcohols **4**–**7**. Reagents and conditions: a) PhCH_2_MgCl (**1**) or PhCH_2_MgBr (**2**) (1.2 equivalents), Et_2_O; see methods A–E in Experimental; b) PDC, DCM, Ac_2_O, reflux, 3 h.

In the context of our studies on the synthesis of sugar amino acids structurally related to iminosugar mannojirimycin (a strong inhibitor of α-mannosidase), we have recently prepared, by applying the Grignard reaction, several branched sugar carbinols as intermediates for subsequent oxidation to ketones affording the corresponding hydantoins (precursors of amino acids) via the Bucherer–Bergs reaction in the next step, which were finally transformed into sugar amino acids (precursors of biologicaly active iminosugars). Thus, using methylmagnesium iodide, some alanine-branched sugars were obtained [9–10]. Analogously, leucine derivatives were synthesised starting from isobutylmagnesium bromide [11]. Unexpected difficulties were encountered in an attempt to prepare the benzyl-branched sugar carbinol, as a precursor of the final phenylalanine-branched sugar, using benzylmagnesium chloride (**1**) or benzylmagnesium bromide (**2**) in the Grignard reaction with 2,3-*O*-isopropylidene-α-D-*lyxo*-pentodialdo-1,4-furanoside (**3**). In this case, the corresponding *o*-tolyl derivative was obtained as the major product instead of the expected benzyl compound (Scheme 1). Therefore, this reaction was subjected to more detailed inspection.

## Results and Discussion

Although **1** and **2** have frequently been used for the introduction of the benzyl group into a carbohydrate molecule [12–18], the benzyl→*o*-tolyl rearrangement has, to the best of our knowledge, only been reported once. In this regard, Panigot and Curley [19] showed that the reaction of **1** with 2,3,4,6-tetra-*O*-acetyl-α-D-glucopyranosyl bromide (or chloride) produced a 3:1 mixture of 2-(β-D-glucopyranosyl)toluene and (β-D-glucopyranosyl)phenylmethane isolated as the corresponding 2,3,4,6-tetra-*O*-acetates. However, this is not a case of the typical Grignard reaction where the Grignard reagent is coupled with a carbonyl compound. Moreover, the anomeric position was involved in the reaction and there was an important limitation: the formation of the unexpected *o*-tolyl rearrangement product entailed the participation of both of the equatorially disposed 2- and 6-acetoxy groups present in the substrate. The non-acetylated substrates (like *O*-benzyl) afforded solely non-rearranged benzyl derivatives. Our results represent the situation where the benzyl→*o*-tolyl rearrangement occurs during the Grignard reaction between **1** or **2** and the non-anomeric free aldehyde function (C-5 position of the furanose) of an *O*-alkyl (methyl and isopropylidene)-protected carbohydrate.

First, the addition reaction of aldehyde **3** with **1** under the standard Grignard reaction conditions (method A, see Experimental part) was examined. Based on the NMR spectral data of the isolated product, a mixture of 5-(*R*) and 5-(*S*) *o*-tolyl derivatives **4** and **5** together with a mixture of 5-(*R*) and 5-(*S*) benzyl derivatives **6** and **7** (Scheme 1) in the ratio of approximately 3:1 was confirmed, indicating the substantial predominance of the benzyl→*o*-tolyl rearrangement. In the subsequent experiments (methods B–E), the influence of the reaction conditions (temperature, reactants ratio, solvent, sequence of reactants addition) was examined, with the aim of suppressing the formation of the rearranged products **4** and **5**. As the preliminary experiments revealed that the addition of a solution of Grignard reagent **2** into a solution of the carbohydrate aldehyde **3** (i.e., reverse addition of reactants) in the mole ratio of 1.3:1 had some positive effect in respect of the yield (but not of the proportion of isomers) of the Grignard reaction products, we applied these parameters in subsequent experiments. It was found that the application of **2** instead of **1** also afforded a mixture of *o*-tolyl and benzyl carbinols but their proportion was dependent on the reaction conditions (see Table 1 and Table 2). The mixture of diastereomeric carbinols **6** and **7** resulted as a main product of the “normal” Grignard reaction only at lower temperature without excess of **2**. Only a minor positive effect on the formation of “normal” addition products **6** and **7** was observed using 2-methyltetrahydrofuran as a solvent as well as the addition of an equivalent of CeCl_3_·2LiCl in THF to the reaction mixture, although it is known that the application of these reagents favours the addition of Grignard reagents to carbonyl compounds to afford higher yields [20–22].

**Table 1:** Reaction conditions for the Grignard reaction.

Method	Entry^a^	Conditions^b^	Conditions^c^

A	**1**/**3** = 3:1	0.5 h, rt	3 h, reflux
B	**2**/**3** = 1.3:1	0.5 h, −25 °C	1.5 h, rt
C	**2**/**3** = 2.5:1	0.5 h, −25 °C	1.5 h, rt
D	**2**/**3** = 1.3:1	0.5 h, rt	2 h, rt
E	**1**/**3** = 1.3:1	0.5 h, −25 °C	1.5 h, rt

^a^Mole ratio of reagent **1** or **2** and aldehyde **3** taken into the Grignard reaction; ^b^time and temperature for addition of reactant **1** or **2** to **3** (in case of method A, reverse addition of reactants was applied, i.e., aldehyde **3** was added to Grignard reagent **1**; ^c^reaction time and temperature after addition of reactants.

**Table 2:** Overall yields of **8** and **9** from oxidation of mixture of carbinols **4**–**7**.

Method	Entry^a^	Overall yield			**8**/**9** ratio

	**4**–**7**	**8** and **9**	Ketone **8**	Ketone **9**	

A	0.62 g	0.59 g, 20%	0.44 g, 15%	0.15 g, 5%	3:1
B	1.37 g	1.29 g, 44%	0.30 g, 10%	0.99 g, 34%	1:3
C	1.17 g	1.11 g, 38%	0.58 g, 20%	0.53 g, 18%	1.1:1
D	0.82 g	0.77 g, 26%	0.56 g, 19%	0.21 g, 7%	2.7:1
E	0.96 g	0.89 g, 30%	0.62 g, 21%	0.27 g, 9%	2.3:1

^a^Amount of mixture of carbinols **4**–**7** taken into oxidation obtained from 10 mmol of aldehyde **3** applying methods A–E.

**Figure 1:**
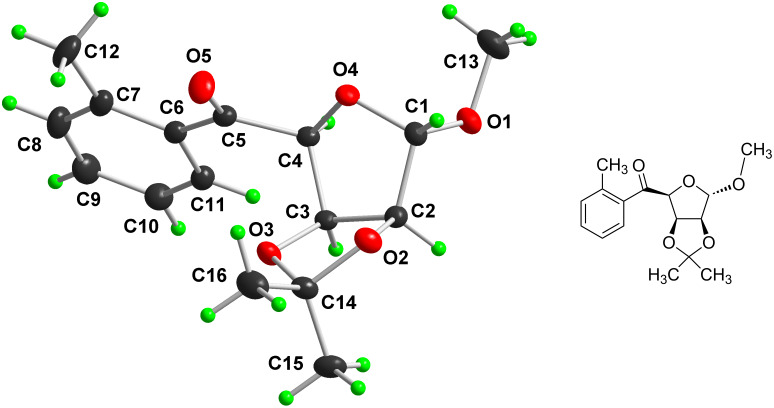
Molecular structure (DIAMOND drawing with adjacent ChemDraw image) of *o*-tolyl derivative **8**. Atomic displacement ellipsoids are drawn at 30% probability level and H atoms are shown as small spheres of arbitrary radii.

**Figure 2:**
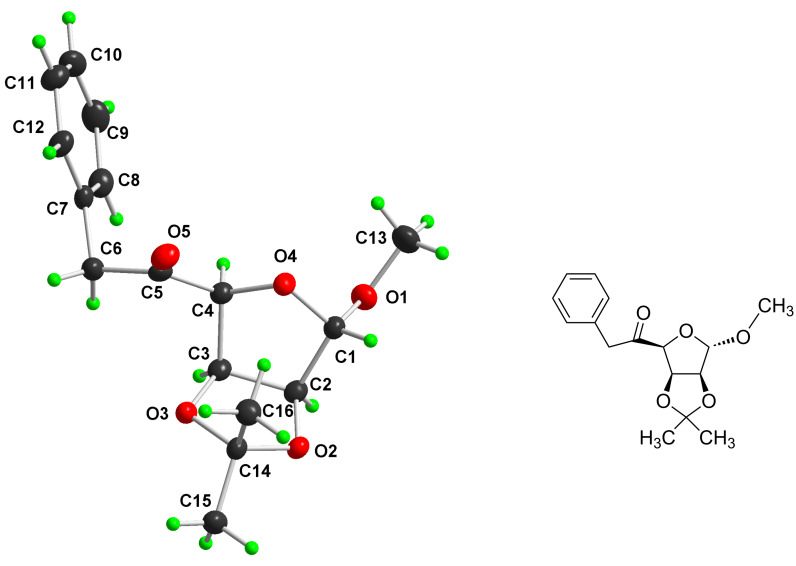
Molecular structure (DIAMOND drawing with adjacent ChemDraw image) of benzyl derivative **9**. Atomic displacement ellipsoids are drawn at 50% probability level and H atoms are shown as small spheres of arbitrary radii.

Since all attempts to separate and purify carbinols **4**–**7** using column chromatography were unsuccessful even after acetylation, their physical and spectral data are not given here in detail. In this regard, the ratio of the *R* and *S* isomers thus formed has not been studied. However, based on the NMR data (signals for methyl and methylene group of *o*-tolyl and benzyl group, respectively) of the isolated crude mixture of products **4**–**7**, it was possible to determine the relative ratio of *o*-tolyl and benzyl isomers. Finally, for the separation of *o*-tolyl isomer from benzyl isomer, a mixture of all four chromatographically non-separable isomeric alcohols **4**–**7** was oxidised using PDC, thereby destroying the chiral center at C-5 position of the saccharide moiety, to afford a mixture of the two corresponding crystalline ketones **8** and **9**. These were successfully separated and purified using column chromatography and recrystallisation. Their structures were established on the basis of ^1^H and ^13^C NMR spectral data. The EI mass spectra and the data of elemental analysis were also confirmative. The singlet signal observed for the H1 atom strongly supports the α-configuration at the anomeric atom C1 with the equatorially positioned H1 and H2. A singlet (three protons) at δ = 3.91 and a singlet (two protons) at δ = 2.48 clearly indicate the methyl (in *o*-tolyl) and methylene (in benzyl) groups, respectively. Finally, the *o*-tolyl structure and benzyl structure of the moieties at C-5 atom of ketone **8** (Figure 1) and ketone **9** (Figure 2), respectively, (the numbering of the atoms is in accordance with the numbering recommended by the IUPAC Nomenclature of Carbohydrates [23]) was unambiguously confirmed by single-crystal X-ray analysis.

**Scheme 2:**
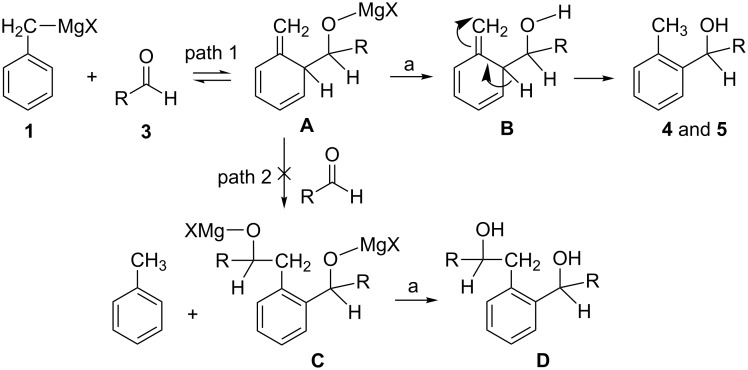
Proposed mechanism for Grignard reaction leading to benzyl→*o*-tolyl rearrangement (path 1). R = saccharide moiety; a = NH_4_Cl, H_2_O.

**Scheme 3:**
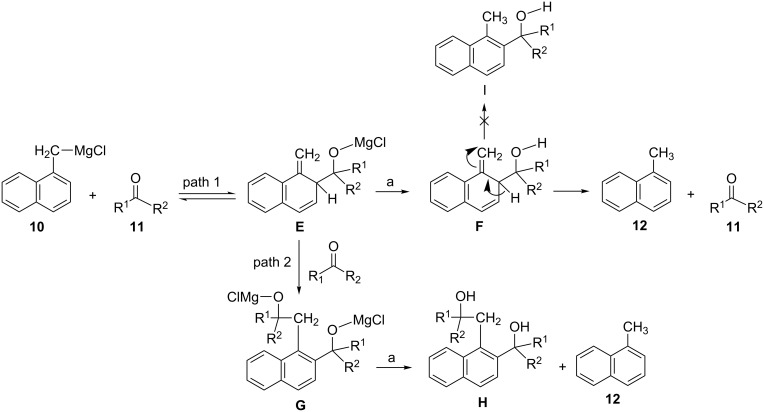
Proposed mechanism [24–25] for Grignard reaction leading to 1-naphthylmagnesiumchloride→1-methylnaphthalene rearrangement. R_1_, R_2_ = H, alkyl, cycloalkyl, phenyl, benzyl; a = NH_4_Cl, H_2_O.

The formation of *o*-tolyl isomers **4** and **5** can be explained by the possible reaction sequence (path 1) depicted in Scheme 2. The first step involves an addition of the Grignard reagent to the saccharide aldehyde, producing a trienic magnesium alkoxide intermediate **A** (magnesium salt of 2-R-hydroxymethyl-1-methylene-1,2-dihydrobenzene, where R is the saccharide moiety) which, upon quenching with aqueous NH_4_Cl, affords (via an intermediate **B**) the corresponding *o*-tolyl isomer (a mixture of diastereomeric alcohols **4** and **5**). A similar mechanism was proposed [24–25] for the reaction of 1-naphthylmethylmagnesium chloride (**10**) with some aldehydes and ketones (Scheme 3). However, in this case, a trienic magnesium alkoxide intermediate **E** (magnesium salt of 2-hydroxymethyl-1-methylene-1,2-dihydronaphthalene, an analogue of intermediate **A** in Scheme 2, path 1), produced by an addition of the Grignard reagent **10** to the monomeric formaldehyde (**11**, R^1^ = R^2^ = H), was unstable and decomposed by a reversible process into the Grignard reagent and aldehyde. The latter underwent a Prins-type reaction with the magnesium alkoxide intermediate **E** in the presence of MgCl_2_, to give magnesium salt **G** which, upon quenching with aqueous NH_4_Cl, affords 1-(2-hydroxyethyl)-2-hydroxymethylnaphthalene **H** (an analogue of diol **D** in Scheme 2, path 2) and 1-methylnaphthalene (**12**). On the other hand, the reaction of **10** with ketones produced either normal benzylic alcohols, rearranged alcohols or a mixture of both, depending on the steric hindrance. However, the rearranged alcohols representing 1-methylene-2-substituted-1,2-dihydronaphthalenes **F** (analogues of intermediate **B** in Scheme 2) were unstable and decomposed to 1-methylnaphthalene (**12**) and the starting ketone **11** without the formation (via an intermediate **F**) of 1-methyl-2-substituted-naphthalenes **I**, analogues of *o*-tolyl derivatives **4** and **5** (Scheme 2, final step of path 1), which were in present study, by contrast, isolated as stable products. Moreover, path 2 in Scheme 2 can be excluded, since no diols of type **C** (analogues of diols **H** in Scheme 3) were detected in the reaction mixture.

To extend and confirm these observations, the analogous addition of **1** as well as **2** to another two representative carbohydrate aldehydes – 3-*O*-benzyl-1,2-*O*-isopropylidene-α-D-*xylo*-pentodialdo-1,4-furanose and 1,2:3,4-di-*O*-isopropylidene-α-D-*galacto*-hexodialdo-1,5-pyranose were investigated next. Unfortunately, only very complex reaction mixtures (including diatereomeric carbinols, biphenyl, polymeric impurities, etc.) resulted under the above reaction conditions. However, analysis of the NMR spectral data of these mixtures showed the absence of CH_3_ protons of the *o*-tolyl moiety, indicating that the benzyl→*o*-tolyl rearrangement did not occur in these cases; accordingly, no further detailed inspections of the reaction mixtures were performed.

### X-ray analysis

Single crystals (stable at ambient temperature) suitable for X-ray diffraction were obtained by the slow crystallisation of **8** and **9** from MeOH by cooling in a refrigerator. The preliminary orientation matrices and final cell parameters were obtained using Siemens SMART and Siemens SAINT software [26]. The data were empirically corrected for absorption and other effects using the SADABS program [27] based on the method of Blessing [28]. The crystal and experimental data for **8** and **9** are summarised in Table 3 and Table 4. The structure was solved by direct methods and refined by full-matrix least-squares on all *F*^2^ data using Bruker SHELXTL [29]. The non-H atoms were refined anisotropically. All the hydrogen atoms were constrained to the geometrically idealised positions using an appropriate riding model. Molecular graphics were obtained using the program DIAMOND [30].

**Table 3:** Crystallographic and experimental data^a^ for compound **8**.

Crystal data	

Empirical formula	C_16_H_20_O_5_
Formula weight	292.32
Crystal size	1.00 x 0.36 x 0.18 mm
Crystal description	Needle
Crystal colour	Colourless
Crystal system	Orthorhombic
Space group	*P*2_1_2_1_2_1_
Unit cell dimensions	*a* = 8.0419(2) Å
	*b* = 10.0156(2) Å
	*c* = 19.2682(3) Å
Volume	1551.95(6) Å^3^
*Z*	4
Calculated density	1.251 Mg/m^3^
Absorption coefficient	0.093 mm^−1^
F(000)	624

Data collection	

Measurement device type	Siemens SMART CCD
Measurement method	ω-scans
Temperature	173(2) K
Wavelength	0.71073 Å
Monochromator	Graphite
θ range for data collection	2.11 to 28.33°
Index ranges	−10 ≤ *h* ≤10, −13 ≤ *k* ≤13, −25 ≤ *l* ≤25
Reflections collected/unique	21019/2217 [*R*(int) = 0.0496]
Completeness to θ = 28.33°	99.7%
Absorption correction	Multi-scan
Max. and min. transmission	0.9835 and 0.9132

Refinement	

Refinement method	Full-matrix least-squares on *F*^2^
Data/restraints/parameters	2217/0/214
Goodness-of-fit on *F*^2^	1.025
Final *R* indices [*I*>2σ(I)]	*R*_1_ = 0.0322, *wR*_2_ = 0.0797
*R* indices (all data)	*R*_1_ = 0.0398, *wR*_2_ = 0.0850
Largest diff. peak and hole	0.198 and −0.149 *e*·Å^−3^

^a^Standard deviations in parentheses.

**Table 4:** Crystallographic and experimental data^a^ for compound **9**.

Crystal data	

Empirical formula	C_16_H_20_O_5_
Formula weight	292.32
Crystal size	0.98 x 0.21 x 0.12 mm
Crystal description	Needle
Crystal colour	Colourless
Crystal system	Monoclinic
Space group	*P*2_1_
Unit cell dimensions	*a* = 10.5035(14) Å
	*b* = 5.7075(8) Å, β = 96.268(3)°
	*c* = 12.3449(16) Å
Volume	735.64(17) Å^3^
*Z*	2
Calculated density	1.320 Mg/m^3^
Absorption coefficient	0.098 mm^−1^
F(000)	312

Data collection	

Measurement device type	Siemens SMART CCD
Measurement method	ω-scans
Temperature	153(2) K
Wavelength	0.71073 Å
Monochromator	Graphite
Theta range for data collection	2.42 to 29.20°
Index ranges	−14 ≤ *h* ≤ 14, −7 ≤ *k* ≤ 7, −16 ≤ *l* ≤ 16
Reflections collected/unique	10030/2175 [*R*(int) = 0.0516]
Completeness to θ = 29.20°	99.4%
Absorption correction	Multi-scan
Max. and min. transmission	0.9884 and 0.9104

Refinement	

Refinement method	Full-matrix least-squares on *F*^2^
Data/restraints/parameters	2175/1/193
Goodness-of-fit on *F*^2^	1.002
Final *R* indices [*I*>2σ(I)]	*R*_1_ = 0.0329, *wR*_2_ = 0.0802
*R* indices (all data)	*R*_1_ = 0.0411, *wR*_2_ = 0.0842
Largest diff. peak and hole	0.188 and −0.195 *e*·Å^−3^

^a^Standard deviations in parentheses.

Based on the calculated values of the ring-puckering parameters (*Q*, Φ, θ) [31] (Table 5) and relevant torsion angles (Table 6), the conformations of the five-membered O4–C1–C2–C3–C4 furanose ring and the five-membered 1,3-dioxolane ring (O2–C2–C3–O3–C14) in compounds **8** and **9** were established. It was found that the furanose ring in **8** adopted the ^O^*E* (^O4^*E*) conformation distorted significantly to the ^O^*T*_1_ (^O4^*T*_C1_, twisted on O4–C1, O4-*endo*) direction. The conformation of the five-membered 1,3-dioxolane ring in **8** can be described as a ^4^*E* (^C14^*E*) shifted significantly to the ^4^*T*_O_ (^C14^*T*_O2_, twisted on C14–O2, C14-*endo*) direction. Regarding compound **9**, an inspection of the relevant data revealed an almost perfect ^O^*E* (^O4^*E*) conformation of the furanose ring and the *E*_4_ (*E*_C14_) conformation distorted significantly to the ^O^*T*_4_ (^O2^*T*_C14_, twisted on O2–C14, C14-*exo*) direction of the 1,3-dioxolane ring.

**Table 5:** Puckering parameters^a^ for the five-membered furanose ring and the five-membered 1,3-dioxolane ring in compounds **8** and **9**.

Ring	Parameter	Compound	

		**8**	**9**

Furanose	*Q* (Å)	0.3467(16)	0.3206(16)
	Φ (°)	8.1(3)	353.8(9)
1,3-Dioxolane	*Q* (Å)	0.3299(16)	0.2146(16)
	Φ (°)	155.1(3)	332.9(4)

^a^Standard deviations in parentheses.

**Table 6:** Relevant torsion angles (°)^a^ for the five-membered furanose ring and the five-membered 1,3-dioxolane ring in compounds **8** and **9**.

Ring	Torsion angle	Compound	

		**8**	**9**

Furanose	O4–C1–C2–C3	26.34(16)	17.62(18)
	C1–C2–C3–C4	−5.02(16)	3.34(18)
	C2–C3–C4–O4	−17.45(15)	−23.03(17)
	C3–C4–O4–C1	35.47(15)	35.73(16)
	C4–O4–C1–C2	−39.06(17)	−33.52(17)
1,3-Dioxolane	O2–C2–C3–O3	−6.58(16)	3.57(18)
	C2–C3–O3–C14	−16.04(16)	11.31(17)
	C3–O3–C14–O2	32.66(16)	−21.76(17)
	O3–C14–O2–C2	−37.19(16)	24.23(17)
	C14–O2–C2–C3	26.66(16)	−17.24(17)

^a^Standard deviations in parentheses.

## Conclusion

In summary, various reaction conditions were employed for the synthesis of carbinols **4**–**7** from Grignard reagent **1** (or **2**) and sugar aldehyde **3**. Depending on the reaction conditions, the ratio of *o*-tolyl carbinols **4** and **5** (products of the benzyl→*o*-tolyl rearrangement) versus non-rearranged benzyl carbinols **6** and **7** varied from 3:1 to 1:3 (based on the isolated *o*-tolyl and benzyl ketones **8** and **9**, respectively). It seems that the benzyl→*o*-tolyl rearrangement is specific for 2,3-*O*-isopropylidene-α-D-*lyxo*-pentodialdo-1,4-furanoside because no rearrangement was observed when 3-*O*-benzyl-1,2-*O*-isopropylidene-α-D-*xylo*-pentodialdo-1,4-furanose and 1,2:3,4-di-*O*-isopropylidene-α-D-*galacto*-hexodialdo-1,5-pyranose were used as starting sugar aldehydes. The structures of *o*-tolyl and benzyl derivatives **8** and **9** were unambiguously confirmed by X-ray crystallographic analysis. Compounds **8** and **9** represent profitable synthetic blocks for the synthesis of structurally modified iminosugars and sugar moiety-containing heterocycles, amino acids, etc.

## Experimental

**Reagents and apparatus:** The starting methyl 2,3-*O*-isopropylidene-α-D-*lyxo*-pentodialdo-1,4-furanoside was prepared according to a recognised method [32–33]. Benzylmagnesium chloride (**1**) and benzylmagnesium bromide (**2**) solutions (1 M in diethyl ether) and the other reagents and solvents were commercially available products and were used as received. Melting points were determined using a Boetius PHMK 05 microscope. Specific rotations were determined on a Jasco P-2000 digital polarimeter. Microanalyses were performed on a Fisons EA 1108 analyser. The ^1^H and ^13^C NMR spectra (in CDCl_3_, internal standard Me_4_Si) were recorded on a Varian 600 MHz VNMRS spectrometer equipped with HCN ^13^C enhanced salt-tolerant cold probe operating at 599.84 and 150.84 MHz working frequencies, respectively. Advanced techniques from the Varian pulse sequence library of 2D homo- and hetero-correlated spectroscopy (gCOSY, gTOCSY, gHSQCAD, gHMBCAD, and gH2BC) including 1D sequences with selective excitations (1DNOESY, 1DTOCSY, and 1DROESY) were used for the signal assignments. The quaternary carbon atoms were identified on the basis of a semi-selective INEPT experiment and a 1D INADEQUATE pulse sequence technique. When reporting assignments of NMR signals, the data for the phenyl moiety are identified by a prime. The EI mass spectra (70 eV) were obtained on a Q-Tof Premier instrument (Waters). Column chromatography was performed as flash chromatography on Silica Gel 60 (E. Merck, 0.063–0.200 mm). The IR spectra (ATR) were measured using a Nicolet 6700 FTIR spectrometer. Thin-layer chromatography was performed on pre-coated Silica Gel 60 F254 plates (E. Merck). Visualisation was achieved by spraying the plates with 5% (v/v) solution of H_2_SO_4_ in ethanol and heating at ca 200 °C.

**Synthesis of methyl (5*R*)-2,3-*O*-isopropylidene-5-*C*-(2-methylphenyl)-α-D-lyxofuranoside (4), methyl (5*S*)-2,3-*O*-isopropylidene-5-*C*-(2-methylphenyl)-α-D-lyxofuranoside (5), methyl 6-deoxy-2,3-*O*-isopropylidene-6-phenyl-α-D-mannofuranoside (6), and methyl 6-deoxy-2,3-*O*-isopropylidene-6-phenyl-β-L-gulofuranoside (7)**

**Method A:** Methyl 2,3-*O*-isopropylidene-α-D-*lyxo*-pentodialdo-1,4-furanoside (**3**) (2.02 g, 10.0 mmol) in diethyl ether (30 mL) was added dropwise into benzylmagnesium chloride (**1**, 1.0 M solution in diethyl ether, 13 mL, 13 mmol) at ambient temperature in the course of 30 min. Subsequently, the mixture was stirred and heated under reflux for 3 h. After cooling, it was poured into a saturated NH_4_Cl solution pre-cooled to 5 °C. The organic layer was separated and the aqueous layer was extracted with diethyl ether (3 × 50 mL). The combined organic layers were dried (Na_2_SO_4_), filtered and evaporated under reduced pressure to give a crude oily product which was purified on a column of silica gel using EtOAc/hexane 1:4 as an eluent, affording a mixture of alcohols **4**–**7** (*R*_f_ = 0.44, 0.62 g, 21%).

**Method B:** Benzylmagnesium bromide (**2**, 1.0 M solution in diethyl ether, 13 mL, 13 mmol) pre-cooled to −5 °C was added dropwise to a magnetically stirred solution of aldehyde **3** (2.02 g, 10.0 mmol) in diethyl ether (30 mL) under cooling at −25 °C in the course of 30 min. Subsequently, the mixture was stirred at ambient temperature for an additional 1.5 h and poured into a saturated NH_4_Cl solution pre-cooled to 5 °C. The organic layer was separated and the aqueous layer was extracted with diethyl ether (3 × 50 mL). The combined organic layers were dried (Na_2_SO_4_), filtered and evaporated under reduced pressure to give a crude oily product which was purified on a column of silica gel using EtOAc/hexane 1:4 as an eluent, affording a mixture of alcohols **4**–**7** (*R*_f_ = 0.44, 1.37 g, 46%).

**Method C:** Benzylmagnesium bromide (**2**, 1.0 M solution in diethyl ether, 25 mL, 25 mmol) and aldehyde **3** (2.02 g, 10.0 mmol) in diethyl ether (30 mL) were allowed to react under the same reaction conditions as in method B to give (after column chromatography) a mixture of alcohols **4**–**7** (*R*_f_ = 0.44, 1.17 g, 39.5%).

**Method D:** Benzylmagnesium bromide (**2**, 1.0 M solution in diethyl ether, 13 mL, 13 mmol) and aldehyde **3** (2.02 g, 10.0 mmol) in diethyl ether (30 mL) were allowed to react at ambient temperature applying the reaction mixture work-up procedure as in method B to give (after column chromatography) a mixture of alcohols **4**–**7** (*R*_f_ = 0.44, 0.82 g, 28%).

**Method E:** Benzylmagnesium chloride (**1**, 1.0 M solution in diethyl ether, 13 mL, 13 mmol) and aldehyde **3** (2.02 g, 10.0 mmol) in diethyl ether (30 mL) were allowed to react under the same reaction conditions as in method B to give (after column chromatography) a mixture of alcohols **4**–**7** (*R*_f_ = 0.44, 0.96 g, 33%).

**Synthesis of methyl 2,3-*O*-isopropylidene-5-*C*-(2-methylphenyl)-α-D-*lyxo*-pentodialdo-1,4-furanoside (8) and methyl 6-deoxy-2,3-*O*-isopropylidene-6-phenyl-α-D-*lyxo*-hexofuranosid-5-ulose (9):** A mixture of alcohols **4**–**7** (1.47 g, 5.0 mmol) in CH_2_Cl_2_ (25 mL) was added dropwise to a magnetically stirred solution of pyridinium dichromate (PDC, 2.45 g, 6.5 mmol) and acetic anhydride (1.3 mL) in CH_2_Cl_2_ (25 mL) under cooling in an ice-water bath followed by heating under reflux for 3 h. The greater part of CH_2_Cl_2_ was evaporated and the chromic salts were precipitated by the addition of a mixture of EtOAc/hexane 1:1. After filtration and evaporation of the solvents under reduced pressure, the product thus obtained was purified on a column of silica gel using EtOAc/hexane 1:4 as an eluent, affording ketone **8** (*R*_f_ = 0.6) and ketone **9** (*R*_f_ = 0.7) with a total yield of 94–96% (percentage of **8** and **9** together in oxidation step). These were recrystallised from methanol. The overall isolated yields of **8** and **9** (depending on the method used for preparation of the starting mixture of alcohols **4**–**7**) from two reaction steps (Grignard reaction and PDC oxidation) starting from 10 mmol of aldehyde **3** are summarised in Table 2.

**8:** colourless solid; mp 105–106 °C; 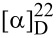 +12 (*c* 1, MeOH); ^1^H NMR (600 MHz, CDCl_3_) δ 7.60–7.22 (m, 5H, Ph), 5.26 (d, *J*_3,4_ = 4.3 Hz, 1H, H-4), 5.12 (s, 1H, H-1), 4.99 (dd, *J*_2,3_ = 5.7 Hz, *J*_3,4_ = 4.3 Hz, 1H, H-3), 4.58 (d, *J*_2,3_ = 5.7 Hz, 1H, H-2), 3.41 (s, 3H, OCH_3_), 2.48 (s, 3H, CH_3_), 1.41 and 1.21 (2s, each 3H, Me_2_C) ppm; ^13^C NMR (150 MHz, CDCl_3_) δ 196.7 (C-5), 138.6 (C-2′), 136.7 (C-1′), 131.8 (C-6′), 131.2 (C-4′), 127.4 (C-3′), 125.3 (C-5′), 113.3 (*C*Me_2_), 107.2 (C-1), 84.4 (C-2), 83.8 (C-4), 81.0 (C-3), 55.1 (OCH_3_), 25.8 and 25.0 [(*C*H_3_)_2_C], 20.3 (CH_3_) ppm; EIMS *m*/*z* (*I*_r_/%): 292 (5), 277 (20), 261 (10), 203 (20), 192 (100), 173 (38), 161 (34), 145 (16), 119 (95), 115 (11), 113 (6), 91 (18), 43 (50); IR (ATR) ν: 2987, 2928, 2834, 1695 (C=O), 1602, 1569, 1487, 1453, 1379, 1278, 1205, 1163, 1110, 1088, 1072, 1011, 964, 922, 886, 856, 825, 784, 761, 729, 659, 647, 619, 591 cm^−1^; anal. calcd (%) for C_16_H_20_O_5_ (292.33): C, 65.70; H, 6.90; found (%): C, 65.56; H, 6.81.

**9:** colourless solid; mp 72–73 °C; 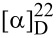 +5 (*c* 1, MeOH); ^1^H NMR (600 MHz, CDCl_3_) δ 7.33–7.21 (m, 5H, Ph), 5.07 (s, 1H, H-1), 4.99 (dd, *J*_2,3_ = 5.7 Hz, *J*_3,4_ = 4.2 Hz, 1H, H-3), 4.56 (d, *J*_2,3_ = 5.7 Hz, 1H, H-2), 4.50 (d, *J*_3,4_ = 4.2 Hz, 1H, H-4), 3.91 (s, 2H, H-6), 3.32 (s, 3H, OCH_3_), 1.45 and 1.28 (2s, each 3H, Me_2_C) ppm; ^13^C NMR (150 MHz, CDCl_3_) δ 203.7 (C-5), 133.5 (C-1′), 129.9 (C-3′, C-5′), 128.5 (C-2′, C-6′), 126.9 (C-4′), 113.1 (*C*Me_2_), 107.6 (C-1), 84.4 (C-4), 84.2 (C-2), 81.0 (C-3), 55.0 (OCH3), 47.1 (C-6), 25.8 and 24.5 [(*C*H_3_)_2_C] ppm; EIMS *m*/*z* (*I*_r_/%): 292 (5), 277 (5), 203 (36), 192 (21), 173 (13), 161 (15), 115 (20), 113 (19), 99 (21), 91 (100), 87 (17), 85 (14), 83 (6), 65 (11), 59 (16), 55 (8), 45 (31), 43 (30), 41 (9); IR (ATR) ν: 2992, 2937, 2868, 2838, 1722, 1600, 1493, 1456, 1433, 1377, 1263, 1238, 1209, 1154, 1121, 1091, 1060, 1029, 996, 968, 874, 798, 753, 720, 699, 658, 611, 560, 513 cm^−1^; anal. calcd (%) for C_16_H_20_O_5_ (292.33): C, 65.70; H, 6.90; found (%): C, 65.52; H, 7.01.

Crystallographic data for structures **8** and **9** have been deposited with the Cambridge Crystallographic Data Centre as supplementary publication nos. CCDC 1001956 and 1001957. Copies of the data can be obtained, free of charge, on application to CCDC, 12 Union Road, Cambridge, CB2 1EZ, UK (fax: + 44-(0)1223-336033 or e-mail: deposit@ccdc.cam.ac.uk or via: http://www.ccdc.cam.ac.uk).
